# Outcomes in patients with endophthalmitis and no light perception vision initially treated with intravitreal antibiotics

**DOI:** 10.1186/s40942-026-00910-6

**Published:** 2026-07-31

**Authors:** Georgia A. Wilke, Christopher Hwang, Henok Getahun, Kenta Kato, Rajendra S. Apte

**Affiliations:** 1 John F. Hardesty, MD Department of Ophthalmology and Visual Sciences, Washington University School of Medicine, St. Louis, MO USA; 2 Retina Consultants of Minnesota, Minneapolis, MN United States; 3 Department of Medicine, Washington University School of Medicine, Box 8096-12-0905, 660 South Euclid Ave, St Louis, MO 63110 USA

**Keywords:** Endophthalmitis, Intravitreal antibiotics, Enucleation, Evisceration, Infection, Microbiology

## Abstract

**Background:**

Management of endophthalmitis presenting with NLP (no light perception) vision remains poorly defined, with limited evidence guiding treatment decisions. We aim to investigate whether treatment with intravitreal antibiotics reduces the rate of enucleation or evisceration of eyes presenting with exogenous or endogenous endophthalmitis and NLP vision.

**Methods:**

Retrospective chart review of patients who presented with endophthalmitis and NLP vision between 2010 and 2020 at a tertiary referral center. Main outcome measures were the proportion of patients receiving intravitreal antibiotics or enucleation/evisceration (ENEV) as initial treatment and the rate of globe preservation in patients who received intravitreal antibiotics.

**Results:**

28 eyes of 26 patients presented with endophthalmitis and NLP vision. The majority of eyes (18/28) were initially treated with intravitreal antibiotics with the aim of globe preservation. Of these 18 eyes, a majority (11/18) achieved globe preservation and the remainder underwent secondary ENEV. Eyes with endogenous and exogenous endophthalmitis that were treated with intravitreal antibiotics achieved similar rates of globe preservation (54% and 57%, respectively). After intravitreal antibiotics, vision in one eye improved to light perception and underwent pars plana vitrectomy to maintain vision. Ultimately, however, the final recorded visual acuity in all eyes was NLP. Additionally, two eyes that received intravitreal antibiotics eventually suffered from phthisical changes, although there was no pain associated with these cases.

**Conclusion:**

In eyes with NLP vision and endophthalmitis, treatment with intravitreal antibiotics leads to a meaningful rate of globe preservation, however the medical decision making in these cases is complex, requiring consideration of the clinical scenario and the patient’s preferences.

## Introduction

Evidence-based management of endophthalmitis is primarily limited to the results of the Endophthalmitis Vitrectomy study (EVS) [1], which examined patients presenting with bacterial endophthalmitis after cataract extraction or secondary intraocular lens implantation. One conclusion of the study was that in patients presenting with LP-only vision, immediate pars plana vitrectomy (PPV) led to a better visual outcome than treatment with intravitreal antibiotics alone. Study participants were limited to patients who had at least LP vision; patients with NLP vision were excluded. There are no studies examining treatment outcomes in eyes with NLP vision and endophthalmitis, therefore there is no standard practice pattern or clear clinical guidance in this situation.

Patients presenting with endophthalmitis and NLP vision may have a higher infection burden and/or more extensive structural damage to the eye [2]. Similarly, patients with NLP vision may be infected by a more virulent pathogen; in EVS, patients with gram-negative infection were more likely to experience severe vision loss than patients with gram-positive, coagulase-negative infection (31% vs. 4%, respectively) [2]. Because of these potential differences, some would argue that endophthalmitis with NLP vision may not be treated effectively with intravitreal antibiotics alone and that ENEV may be necessary for source control. However, ENEV is not without inherent risks. ENEV in cases of endophthalmitis can be associated with significant postoperative complications, such as primary implant exposure or extrusion [3, 4], which then creates the need for additional surgeries and increases the patient’s treatment burden and cost. Moreover, the loss of an eye can cause significant emotional distress to the patient [5] regardless of the eye’s visual potential. Therefore, it is important to determine whether intravitreal antibiotics alone can control infection in this clinical scenario in order to spare the patient from additional morbidity. In this study we describe outcomes of patients presenting with endophthalmitis and NLP vision treated at our institution.

## Methods

This study was approved by the Institutional Review Board of the Washington University School of Medicine and adheres to the tenets of the Declaration of Helsinki. The study design was a retrospective medical chart review of all patients presenting with endophthalmitis in either an inpatient or outpatient setting at Barnes Jewish Hospital between 2010 and 2020. Patients were identified by searching for relevant ICD-9 or ICD-10 codes for endophthalmitis associated with patient encounters. Data collected included demographic information, endophthalmitis etiology, vision on initial exam, pertinent physical exam details such as presence of corneal perforation, presence or absence of orbital spread of infection, types of interventions, and microbiology results. Patients with LP or better vision on initial presentation were excluded. Patients who were intubated and sedated, or mentally altered and unable to give an accurate visual acuity, were also excluded. The primary outcome measures were proportion of patients receiving intravitreal antibiotics or ENEV and rate of globe preservation. Globe preservation was defined as non-removal of the globe even in the presence of phthisical changes.

## Results

There were 336 cases of endophthalmitis identified within the specified time period, with 28 eyes from 26 patients presenting with both endophthalmitis and NLP vision on initial exam. There were 13 eyes with endogenous endophthalmitis (Table 1) and 15 eyes with exogenous endophthalmitis (Table 2). The age range of patients was 31 to 80 years for endogenous endophthalmitis and 42 to 95 years for exogenous endophthalmitis. Overall, there were 14 male patients and 12 female patients. There were two patients in the endogenous group that had bilateral involvement (Table 1, #2 and #10).

**Table 1:** Demographic and clinical information of patients with endogenous endophthalmitis

#	Sex	Age	Initial Treatment	Globe Preservation	Time from dx to ENEV	Orbital involvement	Vision prior to endophthalmitis	Microbiology	Source of systemic infection and other clinical information
1	M	52	IA	Yes	N/A	No	Normal	BCx (+) S. aureus VBx (+) S. aureus (MSSA)	H/o IVDU (+) PPV on day 2 with VBx
2a	F	68	IA	Yes	N/A	Yes	Normal	BCx (+) K. pneumoniae VCx (-)	Liver abscess, (+) DKA
2b	F	68	IA	Yes	N/A	Yes	Normal	BCx (+) K. pneumoniae VCx (-)	Liver abscess, (+) DKA
3	F	46	Evisceration	No	3 days	Yes	Normal	BCx (+) B. cereus Surgical path (+) GMS-positive rods	Uncontrolled DM No source identified
4	F	62	IA	Yes	N/A	No	Normal	BCx (+) S. pneumoniae VCx not sent	Endocarditis
5	M	64	IA	Yes	N/A	No	Normal	BCx (+) P. aeruginosa VCx (+) P. aeruginosa	H/o IVDU, Endocarditis
6	F	71	IA	No	5 days	No	Normal	BCx (+) S. marcescens VCx (+) S. marcescens	Pyelonephritis
7	M	80	Evisceration	No	1 day	Yes	Normal	BCx (+) S. agalactiae Surgical specimen (+) S. agalactiae	Necrotic digit (+) scleral perforation on initial exam
8	M	71	IA	No	5 days	Yes	Normal	BCx (+) E. coli AC fluid (-) growth Surgical specimen (+) E. coli	No source identified
9	M	42	IA	No	3 days	Yes	Normal	BCx (+) B. cereus VCx not sent	H/o IVDU No source identified
10a	F	60	IA + PPV	Yes	N/A	Yes	Normal	BCx (+) S. agalactiae VBx (-)	Endocarditis
10b	F	60	IA	No	12 days	Yes	Normal	BCx (+) S. agalactiae Surgical specimen (+) Gram(+) cocci	Endocarditis (+) scleral abscess on initial exam
11	F	31	IA	No	2 days	Yes	NLP	BCx (+) K. pneumoniae VCx (+) K. pneumoniae	Colonized tunneled line used for HD NLP from PDR and NVG

Abbreviations: Dx = diagnosis, ENEV = enucleation/evisceration, IA = intravitreal antibiotics, N/A = not applicable, BCx = blood culture, VBx = vitreous biopsy, VCx = vitreous culture, PPV = pars plana vitrectomy, NLP = no light perception, PDR = proliferative diabetic retinopathy, NVG = neovascular glaucoma, HD = hemodialysis

**Fig. 1:**
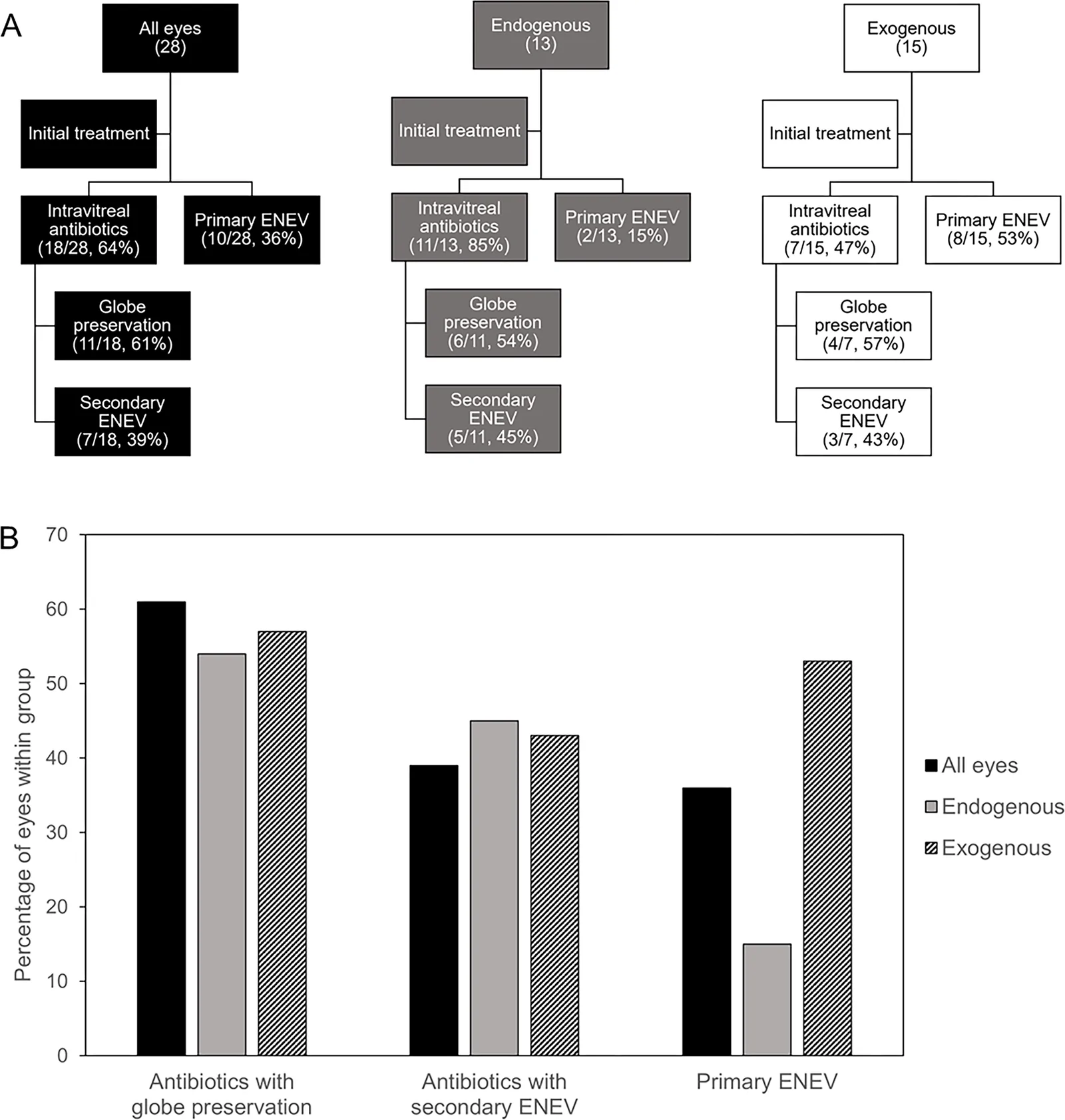
(**A**) Treatment course of patients endophthalmitis. (**B**) Comparison of outcomes in patients with endophthalmitis

In total, 18 eyes (64%) received intravitreal antibiotics as an initial treatment while 10 eyes (36%) underwent primary ENEV (Fig. 1). Of the eyes that received intravitreal antibiotics, 11 achieved globe preservation (61%) while seven underwent secondary ENEV (39%). In the endogenous cases alone, 11 eyes (85%) received intravitreal antibiotics as initial treatment while two eyes (15%) underwent primary evisceration or enucleation. One eye that received intravitreal antibiotics also underwent PPV on the same day as presentation (Table 1, #10a) and another eye underwent PPV on day 2 (Table 1, #1). Patient #10 had bilateral involvement and the decision to undergo PPV was an attempt to salvage at least one globe. Patient #1 improved to LP vision after antibiotic administration, therefore PPV was part of aggressive management to maintain vision but the visual acuity declined to NLP again after surgery. Of the eyes that received intravitreal antibiotics, six eyes achieved globe preservation (54%) while five eyes underwent secondary ENEV (45%). The time from diagnosis to ENEV ranged from 1 to 12 days.

In the exogenous cases alone, seven eyes (47%) received intravitreal antibiotics as initial treatment while eight eyes (53%) underwent primary evisceration or enucleation. One eye that received intravitreal antibiotics also underwent PPV on day two (Table 2, #3) with the explicit goal of “saving the eye” without the expectation of improving vision. Of the eyes that received intravitreal antibiotics, four eyes achieved globe preservation (57%) while three eyes underwent secondary ENEV (43%). The time from diagnosis to ENEV ranged from 1 to 16 days.

**Table 2:** Demographic and clinical information of patients with exogenous endophthalmitis

#	Sex	Age	Initial Treatment	Globe Preservation	Time from dx to ENEV	Orbital involvement	Vision prior to endophthalmitis	Microbiology	Etiology of endophthalmitis and other clinical information
1	M	42	IA	No	3 days	Yes	Normal	VCx not sent Surgical specimen (-) organisms	Self-sealing penetrating injury
2	M	56	Evisceration	No	3 days	Yes	Normal	Ocular swab (+) S. pneumoniae	Blebitis
3	F	85	IA	Yes	N/A	No	Normal	VCx (-)	Blebitis (+)PPV on day 2
4	M	91	Evisceration	No	1 day	No	NLP	Surgical specimen with Gram(+) cocci in chains	Perforated K ulcer NLP from end-stage glaucoma
5	M	56	Evisceration	No	2 days	No	NLP	Corneal swab (+) K. pneumoniae Surgical specimen with Gram(-) bacteria	K ulcer NLP from congenital glaucoma, prosthesis in fellow eye
6	F	95	IA	Yes	N/A	No	NLP	VCx (-)	Blebitis NLP from end-stage glaucoma
7	M	72	Evisceration	No	2 days	No	NLP	Corneal swab (+) P. aeruginosa	K ulcer with impending perforation NLP from distant h/o trauma
8	M	65	IA	Yes	N/A	No	NLP	VCx (-)	K ulcer NLP from h/o compressive optic neuropathy
9	M	72	IA	Yes	N/A	No	NLP	VCx (-)	Blebitis NLP from prior episode of bleb-related endophthalmitis
10	F	74	IA	No	16 days	No	NLP	VCx (-) Surgical specimen (-) organisms	Blebitis NLP from end-stage glaucoma
11	F	94	Evisceration	No	2 days	No	NLP	Corneal swab (+) MSSA	K ulcer NLP from CRVO
12	F	83	Evisceration	No	2 days	No	LP	Corneal swab (+) P. aeruginosa	K ulcer LP from uveitic glaucoma
13	M	86	Evisceration	No	1 day	No	HM/LP	Surgical pathology with Gram(+) cocci	K ulcer (+) perforation Poor vision due to h/o SO after penetrating injury to fellow eye
14	M	59	Enucleation	No	10 days	No	CF	Surgical specimen (-) organisms	Blebitis CF from end-stage glaucoma
15	F	57	IA	Yes	N/A	Yes	LP	VCx (-)	Post-surgical 1 week s/p PPV for TRD repair (history of bilateral PDR)

Abbreviations: Dx = diagnosis, ENEV = enucleation/evisceration, IA = intravitreal antibiotics, N/A = not applicable, BCx = blood culture, VCx = vitreous culture, PPV = pars plana vitrectomy, PDR = proliferative diabetic retinopathy, NVG = neovascular glaucoma, CRVO = central retinal vein occlusion, SO = sympathetic ophthalmia, TRD = tractional retinal detachment, NLP = no light perception, LP = light perception, HM = hand motion, CF = counting fingers

The most common pathogens from the blood cultures of cases of endogenous endophthalmitis were *K. pneumoniae* (3 cases), *S. agalactiae* (3 cases), and *B. cereus* (2 cases) (Table 1). In the majority of endogenous cases, there was no vitreous culture either because there was no fluid return upon vitreous aspiration, aspiration was not attempted (6 cases), or there was no culture growth (3 cases) (Table 1). Microbiologic cultures from exogenous cases, which included vitreous cultures as well as corneal swabs in cases of corneal ulcers, most often yielded no growth (6 cases) or were not performed or the vitreous aspiration was dry (4 cases) (Table 2). Identified pathogens were *P. aeruginosa* (2 cases), *K. pneumoniae* (1 case), *S. pneumoniae* (1 case), and *S. aureus* (1 case) (Table 2). Exogenous cases were most often due to corneal ulcer (7 cases) and blebitis (6 cases) (Table 2). There was one patient who presented several days after an apparently self-sealing penetrating injury (Table 2, #1) and one patient who was one week post-PPV for tractional retinal detachment (TRD) repair (Table 2, #15).

**Fig. 2:**
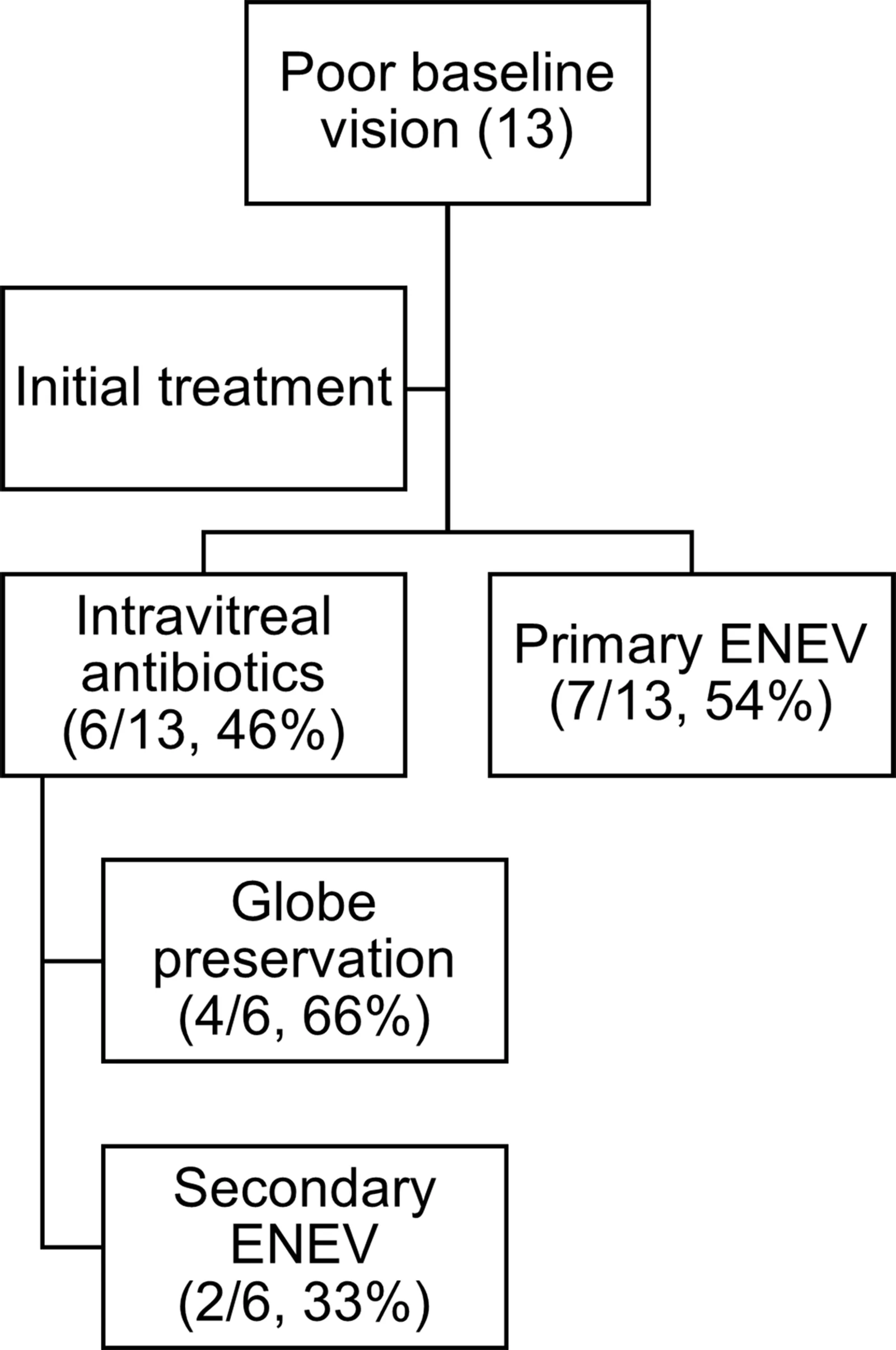
Outcomes of patients with poor baseline vision

The majority of endogenous patients reported “normal” vision prior to the development of endophthalmitis; one patient had baseline NLP vision in the affected eye due to proliferative diabetic retinopathy and neovascular glaucoma (Table 1). The majority of exogenous patients had poor baseline vision, with 12 patients having “counting fingers” (CF) or worse visual acuity (Table 2). Most of the poor vision was due to severe glaucoma (6 patients, Table 2). Of the 13 patients that had poor baseline vision, six received intravitreal antibiotics as initial treatment (46%) and seven underwent primary ENEV (54%) (Fig. 2). Of those that received antibiotics, four achieved globe preservation (66%) and two underwent secondary ENEV (33%) (Fig. 2). Of the 15 eyes from 13 patients who reported having normal vision before endophthalmitis, 12 (80%) were treated with intravitreal antibiotics, one in conjunction with PPV. Of these eyes, seven (58%) achieved globe preservation and five (42%) underwent secondary ENEV (Tables 1 and 2).

**Fig. 3:**
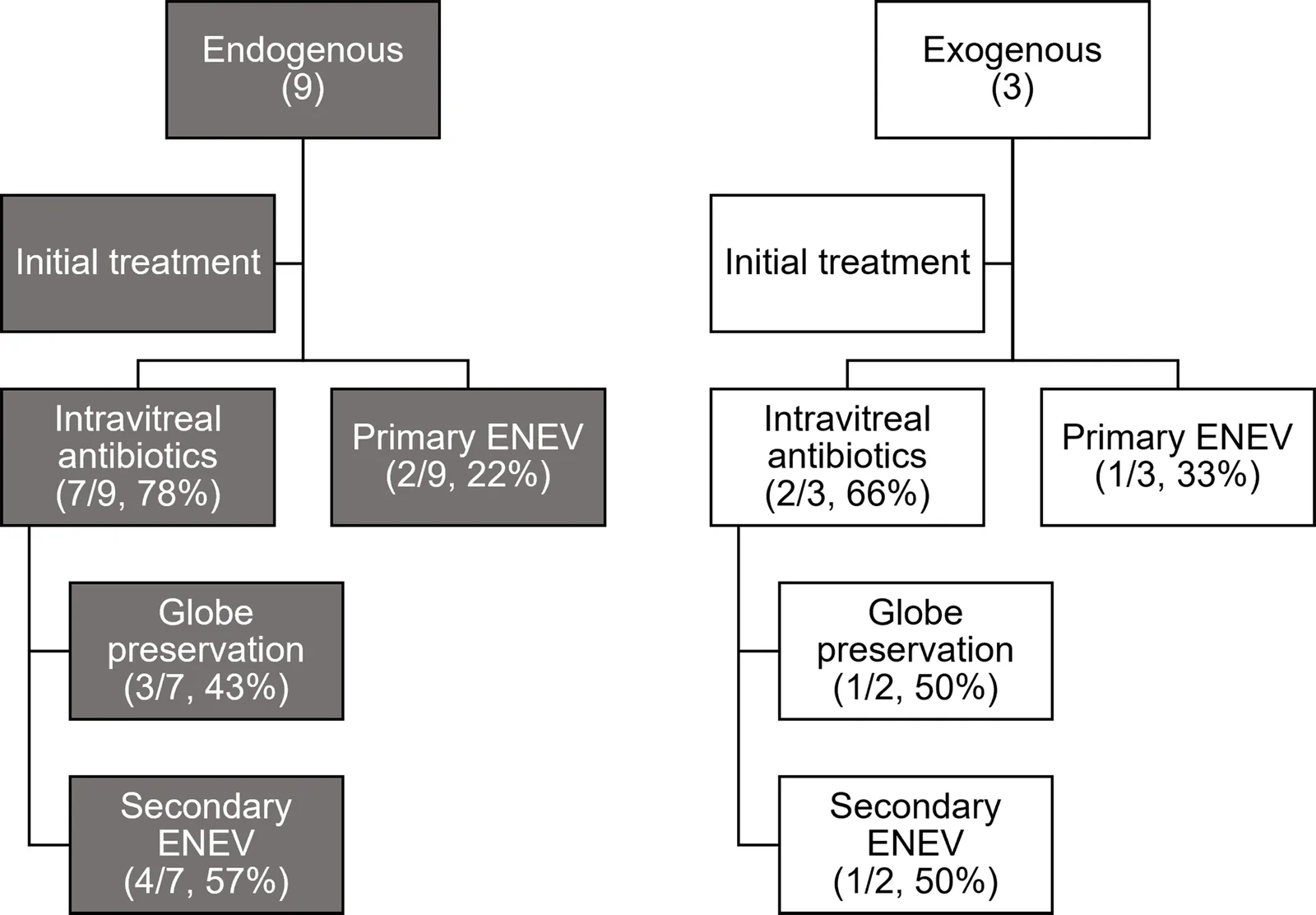
Outcomes of patients with orbital involvement in endogenous and exogenous endophthalmitis

In the endogenous group, nine patients had evidence of orbital involvement (i.e., cellulitis) on exam as well as CT imaging. Of these patients, seven received intravitreal antibiotics as initial therapy (78%) and two underwent primary ENEV (22%) (Fig. 3). Of those that received antibiotics, three achieved globe preservation (43%) and four underwent secondary ENEV (57%). In the exogenous group, three patients had orbital involvement, with two patients receiving intravitreal antibiotics (66%) and one patient undergoing primary ENEV (33%). Of the patients who received antibiotics, one achieved globe preservation (50%) while one underwent secondary ENEV (50%).

Patients who did not undergo ENEV had variable follow-up with ophthalmology after treatment with intravitreal antibiotics. Patients with endogenous endophthalmitis had their last documented ophthalmologic exam ranging from three days to seven months post-diagnosis of endophthalmitis (Table 3). One patient (Table 1, #2) had phthisical changes on exam at seven months post-treatment but denied pain (Table 3). Patients with exogenous endophthalmitis had their last documented ophthalmologic exam ranging from six days to four years post-diagnosis (Table 3). One patient (Table 2, #3) had phthisical changes at four years post-treatment but denied pain. All eyes remained NLP at their last exam (Table 3).

**Table 3:** Follow-up information for patients with endophthalmitis and globe preservation

**Endogenous endophthalmitis**			
**Patient #**	**Last documented exam**	**Final vision**	**Phthisical changes noted?**
1	5 days post-diagnosis, performed while still inpatient, no documentation of outpatient follow-up (lived 3 h away)	NLP	No
2 (a, b)	7 months post-diagnosis	NLP	Yes, but patient comfortable
4	3 days post-diagnosis, performed while still inpatient, no documentation of outpatient follow-up (lived 5 h away)	NLP	No
5	2 weeks post-diagnosis, no records of outpatient follow-up (established with ophthalmology at local VA hospital)	NLP	No
10 (a)	6 weeks post-diagnosis, lived 6 h away	NLP	No
**Exogenous endophthalmitis**			
**Patient #**	**Last documented exam**	**Final vision**	**Phthisical changes noted?**
3	4 years post-diagnosis	NLP	Yes, but patient comfortable
6	3 years post-diagnosis	NLP	No
8	3 months post-diagnosis	NLP	No
9	3 years post-diagnosis	NLP	No
15	6 days post-diagnosis, performed while still inpatient, no documentation of outpatient follow-up (lived 3 h away)	NLP	No

## Discussion

This study describes the outcomes of patients presenting with NLP vision and endophthalmitis. In both endogenous and exogenous cases of endophthalmitis, 50–60% of patients who received intravitreal antibiotics achieved globe preservation (Fig. 1). The 61% rate of globe preservation overall suggests that ENEV can be avoided in a significant proportion of these cases (Fig. 1). In studies reporting endophthalmitis outcomes, primary ENEV is commonly performed in cases with no visual potential [6].– [9] It is unclear in these studies if the decision to remove the eye is based solely on the presence of NLP vision or if other factors, such as orbital spread of infection, are considered. Studies identifying risk factors for ENEV in endophthalmitis report initial visual acuity as an independent risk factor for ENEV [8, 10], but again it is unclear if poor initial visual acuity is indicative of a more virulent infection that requires ENEV for source control or if poor visual potential simply lowers the threshold for considering ENEV.

Primary ENEV should not be seen as an “easier” option in this clinical situation for multiple reasons. Surgery always carries both procedure- and anesthesia -associated risks. Many of these patients, particularly in the endogenous group, had multiple comorbidities that would increase the likelihood of complications with general anesthesia. Additionally, ENEV in cases of endophthalmitis can cause issues with primary implant placement [3, 4], such that many oculoplastic surgeons will delay implant placement [11], though practice patterns vary across institutions [12, 13]. In this study, only two out of the 17 patients who underwent ENEV received primary implant placement (Table 2, #2 and #14). Secondary implant placement obligates the patient to a second surgery, additional hospital and recovery time, as well as increased expense. One retrospective series identified high rates of complications in secondary implant placement [14], which would also create a significant burden for the patient. The patient’s burden also includes pain, with enucleation/orbital implant placement associated with considerable discomfort [15]. Poor post-operative pain control is a major cause of with hospital admission after ambulatory surgery [16, 17], creating an additional cost.

Eyes that have survived a major insult such as endophthalmitis can develop phthisical changes, such as an opaque cornea or shrunken appearance. This understandably can be cosmetically bothersome to the patient and does not necessarily represent a better outcome than eye removal simply because the eye itself was preserved. In this study population, follow-up with ophthalmology was variable (Table 3), so some patients did not have serial exams over a long enough time to say with confidence whether or not the patients developed phthisis bulbi or an otherwise cosmetically unfavorable outcome. Many of these patients traveled from hours away and it was not feasible for them to continue to receive care at our institution. There were two patients who had documented phthisical changes at their last follow-up visit but both denied pain (Table 3). One of these patients was referred to an ocularist for scleral shell fitting, which provides a solution to the issue of cosmetic appearance. Patients who avoid ENEV but develop phthisis can still receive the cosmetic benefit of a prosthesis with a scleral shell. Therefore, the decision to pursue primary ENEV in cases of endophthalmitis and NLP vision should not be made on the basis of future prosthetic options alone.

As mentioned, orbital spread of infection could be considered a deciding factor for ENEV. Orbital involvement of infection was identified in nine patients with endogenous endophthalmitis and three patients with exogenous endophthalmitis (Fig. 3). Studies of concurrent orbital cellulitis and endophthalmitis are mostly limited to case reports [18]– [21] with varying outcomes, with one retrospective study demonstrating good results in terms of globe preservation with intravitreal and periocular injections of antibiotics and steroids. [22] In the current study, several patients with endogenous endophthalmitis and orbital cellulitis were initially treated with intravitreal antibiotics because the patient was systemically unstable and therefore unable to go to the operating room for urgent ENEV. While in these cases it was assumed that intravitreal antibiotics would likely be a temporizing measure, about 40–50% (Fig. 3) of the patients achieved globe preservation. Therefore, presence of orbital involvement of infection should not be considered the sole reason to perform primary ENEV.

During data collection, two groups of patients were identified: patients who had no vision issues prior to the development of endophthalmitis and patients who had poor baseline vision, defined as CF or less. Patients with exogenous endophthalmitis were more likely to have poor baseline vision than patients in the endogenous group (80% vs. 7.6%). It is likely that patients with poor baseline vision are unable to notice early symptoms of infection from either a corneal ulcer or blebitis, the two most common causes of endophthalmitis in this group, and the infection then progressed to endophthalmitis. It has been reported previously that patients with corneal ulcer-associated endophthalmitis have high rates of severe pre-existing ocular pathology,^23^ and this study reinforces that observation. Clearly, patients presenting with endophthalmitis and baseline NLP vision are different than patients who present with fulminant endophthalmitis and new-onset NLP vision. However, one could argue that patients with poor baseline vision and endophthalmitis may have a high infection burden from the delay in seeking care due to lack of recognition of early symptoms, and therefore the question of whether intravitreal antibiotics alone are sufficient to treat the infection is still relevant. It is also important to acknowledge how poor baseline vision affects clinical decision-making in these scenarios – notably, the exogenous group had a higher rate of primary ENEV than the endogenous group (53% vs. 15%). In some cases, intravitreal antibiotics were not an option due to a perforated corneal ulcer (Table 2, patients #4 and #13). However, in other cases, the patient elected for primary ENEV because the affected eye had been causing chronic discomfort prior to infection and the patient had already accepted the loss of useful vision in the eye. Regardless, in patients who received intravitreal antibiotics, there was a reasonable rate of globe preservation (66%, Fig. 2), again demonstrating the utility of the procedure in terms of avoiding surgery.

Certain pathogens, such as *Klebsiella pneuomoniae* and *Streptococcus spp*, have been associated with a more virulent course and worse outcomes in endophthalmitis [6, 22, 24]. It is difficult to comment on outcomes associated with specific pathogens in this study because of the small number of patients as well as the frequent negative microbiologic cultures (Tables 1 and 2). However, the pathogens identified in this study are similar to the results of other retrospective studies examining endogenous [22] and exogenous [23] endophthalmitis. For example, *K. pneumoniae* and *S. agalactiae* were the most common isolates from blood cultures in the endogenous endophthalmitis cases (Tables 1 and 2) and both are known to be associated poor final visual acuity [24, 25].

There are several limitations to this study, including its retrospective nature and small study population. However, given that the study was performed at a tertiary referral center with a large catchment area and only 26 patients over a 10-year period were identified as fitting the criteria, this clinical question is unlikely to be amenable to large, prospective study. The 26 patients included in the study represented a wide variety of clinical situations, which makes it difficult to create an algorithm for treatment of these patients. However, the inability to make generalized guidelines highlights that this specific patient presentation is a unique scenario that requires consideration of the entire clinical context, including the patient’s systemic morbidities, the patient’s desire for globe salvage, etc., when making a treatment recommendation. This study identifies several factors that the physician can consider when deciding treatment, such as the presence orbital spread of infection and the quality of baseline vision prior to the development of endophthalmitis.

Another limitation to this data set is the inclusion of three patients who underwent PPV as part of treatment in addition to intravitreal antibiotics (Table 1, patients #1 and #10; Table 2, patient #3); these patients avoided ENEV. It is unclear why these specific patients underwent PPV when they had similar presentations to other patients in the study who did not receive PPV. Typically, this intervention is reserved for at least LP vision based on the results of EVS [1], though individual practice patterns may vary. This inconsistency highlights the lack of evidence-based treatment guidelines in this patient population.

Endophthalmitis presenting with NLP vision is a difficult clinical situation where the initial treatment choice is not always clear. This study identified two scenarios: endophthalmitis in patients with new-onset NLP vision and patients with baseline poor vision. While the NLP vision may have different clinical significance in these two situations, it still has a large impact on clinical decision making in both scenarios. Lack of vision potential may push the physician and patient towards ENEV, but as this study shows, initial treatment with intravitreal antibiotics can lead to a meaningful rate of globe preservation and spare the patient from eye removal. Primary ENEV in these cases is not a *fait accompli*; treatment of endophthalmitis and NLP vision requires shared decision-making between the physician and patient.

## Data Availability

Data is available in Tables 1, 2 and 3.

## Abbreviations

EVS: Endophthalmitis vitrectomy study
PPV: Pars plana vitrectomy
NLP: No light perception
ENEV: Enucleation/evisceration
LP: Light perception
CF: Counting fingers
